# Urologic Review

**Published:** 1916-02

**Authors:** 


### ABSTRACTS.

Have you ever felt that this department of the BUFFALO MEDICAL JOURNAL was lacking in abstracts of your specialty, or that such as were published lacked the expert judgement which you yourself could have exercised in selection and preparation? If not, you are more charitable than the editor has been to himself. If so, will you assist in abstracting from a few journals, and thus make this department more representative and more valuable? Incidentally, there are few forms of study more helpful to the worker than this.

**Urologic Review.** Chas. W. Bethune. Owing to the fact that the X-ray so frequently fails to demonstrate calculi in the ureter the use of the wax-tipped catheter is advocated. Gearaghty, (Surg. Gyn. & Obs. May, 1915) threads the cystoscope over the catheters which have been passed into the bladder in the same manner as passing a tunneled sound over a filliform. Harris (Trans. Amer. Urolog. Assn. 1914) guards the wax tipped catheter with a small rubber tube during its passage through the catheter channel of a Garceau type cystoscope.

The writer wishes to report a case in which the X-ray failed to demonstrate a calculus the size of a dried pea whose spicules scratched the varnish of a catheter. This stone passed naturally fifteen days later.

